# Sivelestat for septic patients with acute respiratory distress syndrome: a systematic review and meta-analysis of a deadly duo

**DOI:** 10.3389/fmed.2025.1679717

**Published:** 2025-10-20

**Authors:** Wen-He Zheng, Yan-Ge Hu, Da-Xing Yu, Hui-Bin Huang

**Affiliations:** ^1^Department of Critical Care Medicine, The Second People’s Hospital Affiliated to Fujian University of Traditional Chinese Medicine, Fuzhou, China; ^2^Department of Critical Care Medicine, Guang’anmen Hospital, China Academy of Chinese Medical Sciences, Beijing, China

**Keywords:** sivelestat, neutrophil elastase inhibitors, acute respiratory distress syndrome, mortality, meta-analysis

## Abstract

**Background:**

Acute respiratory distress syndrome (ARDS) is one of the most common organ dysfunctions in sepsis. The potential benefits of sivelestat, a selective inhibitor of neutrophil elastase, for patients with septic ARDS remain unclear. The current systematic review and meta-analysis aimed to evaluate the effectiveness of sivelestat in reducing mortality and improving other important outcomes in this patient population.

**Methods:**

We searched PubMed, EMBASE, and Cochrane Library databases until May 30, 2025, for studies comparing sivelestat in septic patients with ARDS against controls. The primary outcome was mortality. We assessed study quality and conducted subgroup analyses, sensitivity analyses, regression analyses, and GRADE evaluations to explore potential heterogeneity.

**Results:**

A total of 17 studies involving 5,062 patients met the inclusion criteria. Overall, sivelestat significantly reduced the risk of mortality compared to controls (odds ratio [OR] = 0.63; 95% confidence interval [CI], 0.48–0.84; *I*^2^ = 39%). Meta-regression showed that differences in baseline PaO_2_/FiO_2_ and risk of mortality significantly influence the effectiveness of sivelestat interventions, as shown in sequent subgroup analyses of patients with partial pressure of oxygen/fraction of inspiration oxygen (PaO_2_/FiO_2_) < 200 mmHg (OR = 0.61; 95% CI 0.51–0.73) and those with a mortality rate greater than 30% (OR = 0.48; 95% CI 0.37–0.60). A similar result was found when we pooled results from adjusted regression analyses (hazard ratio = 0.48; 95% CI 0.28–0.82). Additionally, sivelestat significantly improved PaO_2_/FiO_2_ on days 1, 3, 5, and 7 after treatment and was associated with a significant reduction in the duration of mechanical ventilation (standardized mean difference [SMD] = −0.58 days; 95% CI, −0.96 to −0.19), and length of ICU stay (SMD = −0.76 days; 95% CI, −1.09 to −0.43).

**Conclusion:**

Sivelestat significantly increased PaO_2_/FiO_2_ levels after treatment, leading to a improvement in mortality and other clinical outcomes. Further studies with well-designed protocols for administering sivelestat are needed to validate these findings.

**Systematic review registration:**

https://inplasy.com/wp-content/uploads/2025/06/INPLASY-Protocol-7969.pdf, identifier INPLASY202560111.

## Introduction

Sepsis is a severe condition defined as organ failure due to infection and is associated with high mortality rates (1, 2). Acute respiratory distress syndrome (ARDS) is one of the most common organ dysfunctions during sepsis onset and is characterized by severe hypoxemia, diffuse pulmonary infiltrates and non-cardiogenic pulmonary edema (3–5). Studies have shown that more than half of intensive care unit (ICU) admissions for sepsis result in the development of ARDS (6). Despite significant advances in protective mechanical ventilation (MV) strategies, treatment options for septic ARDS remain limited, resulting in poor prognosis (7). Previous clinical studies have demonstrated that septic patients with high plasma neutrophil elastase (NE) levels are highly susceptible to ARDS (8) because NE from neutrophils induces damage to the vascular endothelium and increases vascular permeability (9). This mechanism is demonstrated as important in the development and progression of ARDS.

Sivelestat is a selective NE inhibitor that belongs to the secondary generation (10). Early animal studies indicated that sivelestat sodium lowers serum levels of interleukin-1β and tumor necrosis factor-*α* and decreases infiltration and activation of inflammatory cells in septic animals (11). Research has also revealed that sivelestat can mitigate and prevent tissue ischemia and reperfusion injury across multiple organs. However, the effectiveness of sivelestat in treating ARDS patients remains controversial. An early multi-center randomized controlled trial (RCT) (12) and two meta-analyses suggested that ARDS did not benefit from sivelestat treatment (13, 14). The high heterogeneity caused by the inclusion of various critically ill populations with different disease severities, among others, may explain these negative results. Consequently, clinical recommendations have emerged, advocating for population-specific treatment of ARDS with sivelestat. In 2020, the State Administration of Pharmaceutical Products of China approved sivelestat sodium for entry into China to treat patients with septic ARDS (Drug Administration Code: H20203093). Several published studies have reported that sivelestat improves oxygenation, decreases the duration of MV, and length of stay (LOS) in ICU, and even reduces total costs in patients with septic ARDS (15–18). However, there is still a lack of high-level evidence for the sivelestat treatment in this patient population.

Recently, several studies on this topic have emerged (15, 16, 18–20). Therefore, we aimed to conduct a systematic review and meta-analysis to investigate the effects of sivelestat on the clinical outcomes of ARDS patients with sepsis. We hypothesize that sivelestat could benefit this patient population and further explore potential factors influencing these outcomes.

## Methods

The protocol for the current review was registered with the International Platform of Registered Systematic Review and Meta-analysis Protocols database (registered number: INPLASY202560111). We conducted our study adhered to the Preferred Reporting Items for Systematic Reviews and Meta-Analysis (PRISMA) statement (21)(Supplementary file 1).

### Search strategy and data sources

Two authors conducted an independent electronic search in PubMed, Embase, Web of Science, and the Cochrane Library up to May 30, 2025 without any restrictions on publication type or language. The search strategy included Medical Subject Headings (MeSH) and keyword terms (“sivelestat,” or “neutrophil elastase inhibitor,” or “ONO 5046,”) and (“ARDS,” or “acute respiratory distress syndrome,” or “acute lung injury”) and (“septic,” or “sepsis”). To ensure that no relevant literature was overlooked, we also reviewed the reference lists of included studies, previously published reviews, and expert opinions.

### Study selection and inclusion criteria

The current meta-analysis incorporated studies that fulfilled the following PICOS criteria: (i) Participants: adult patients aged 18 years or older who have been diagnosed with sepsis and ARDS/ALI, based on the diagnostic definitions established for each study period (7, 22, 23). (ii) Interventions: sivelestat therapy. (iii) Comparisons: standard care or placebo. (iv) Outcomes: all-cause mortality, length of stay in ICU and hospital, duration of MV, complications, and partial pressure of oxygen/fraction of inspiration O_2_ (PaO_2_/FiO_2_) levels; and (v) Study design: RCTs or observational studies with two or more arms.

We excluded the studies as follows: (1) studies enrolled patients younger than 18 years old, pregnant women, or breastfeeding women; (2) publications in abstract, meeting reports and reviews; and (3) studies without reporting any predefined outcomes. In addition, when multiple studies reported the same cohort, the study with the largest sample size with relevant outcome data was included.

### Data extraction

Two authors independently extracted relevant information from eligible studies. The extracted data included study characteristics (first author’s name, study period, and publication year), patient demographics (age, gender, patient population, disease severity, body mass index), details about the sivelestat and control protocols, predefined outcomes, and data for study quality assessment.

### Outcomes

The primary outcomes were mortality at the longest follow-up available. Secondary outcomes included important clinical outcomes, such as ICU and hospital length of stay, duration of mechanical ventilation, arterial oxygen pressure to fraction of oxygen (PaO_2_/FiO_2_) level on baseline, and 1, 3, 5, and 7 days after treatment, serum neutrophil elastase concentrations, and adverse events (defined by each study author).

### Quality assessment

The Cochrane Risk-of-Bias (ROB), a tool developed by Cochrane for RCTs, was used to assess the quality of each study (24). Visual inspection funnel plots were used to evaluate publication bias when a minimum of 10 studies were included in the meta-analysis. Discussion and consensus were used to resolve disagreements.

### Statistical analysis

We estimate the pooled odds ratios (ORs) and mean differences (MDs) with associated 95% confidence intervals (CIs) for dichotomous and continuous variables, respectively. For studies reporting the median with an interquartile range (IQR) as treatment effect measure, we converted median to mean and IQR to standard deviations (SD) according to the Cochrane methods (25). The *I*^2^ statistic and Cochran’s Q test were used to test for heterogeneity, with *I*^2^ values interpreted as 0–30% (not important), 30–60% (moderate), 60–90% (substantial), and > 90% (considerable) (26). A fixed-effect model was used when *I*^2^ < 30%, and a random-effect model was used when *I*^2^ > 50%, using the Mantel–Haenszel method.

To investigate the potential influencing factors, we performed sub-group analyses based on several ARDS related clinical variables, including the PaO_2_/FiO_2_ (initial <200 mmHg vs. <300 mmHg), mortality rate (<30% vs. ≥30%), sample size (<100 vs. ≥100), and study design (RCT vs. non-RCT). We used random-effects meta-regression to explore the possible source of heterogeneity. In addition, sensitivity analyses were performed by sequentially excluding each study to assess the robustness of the results. The significance level for *p* values was set at 0.05.

## Results

### Searching results

**Figure 1:**
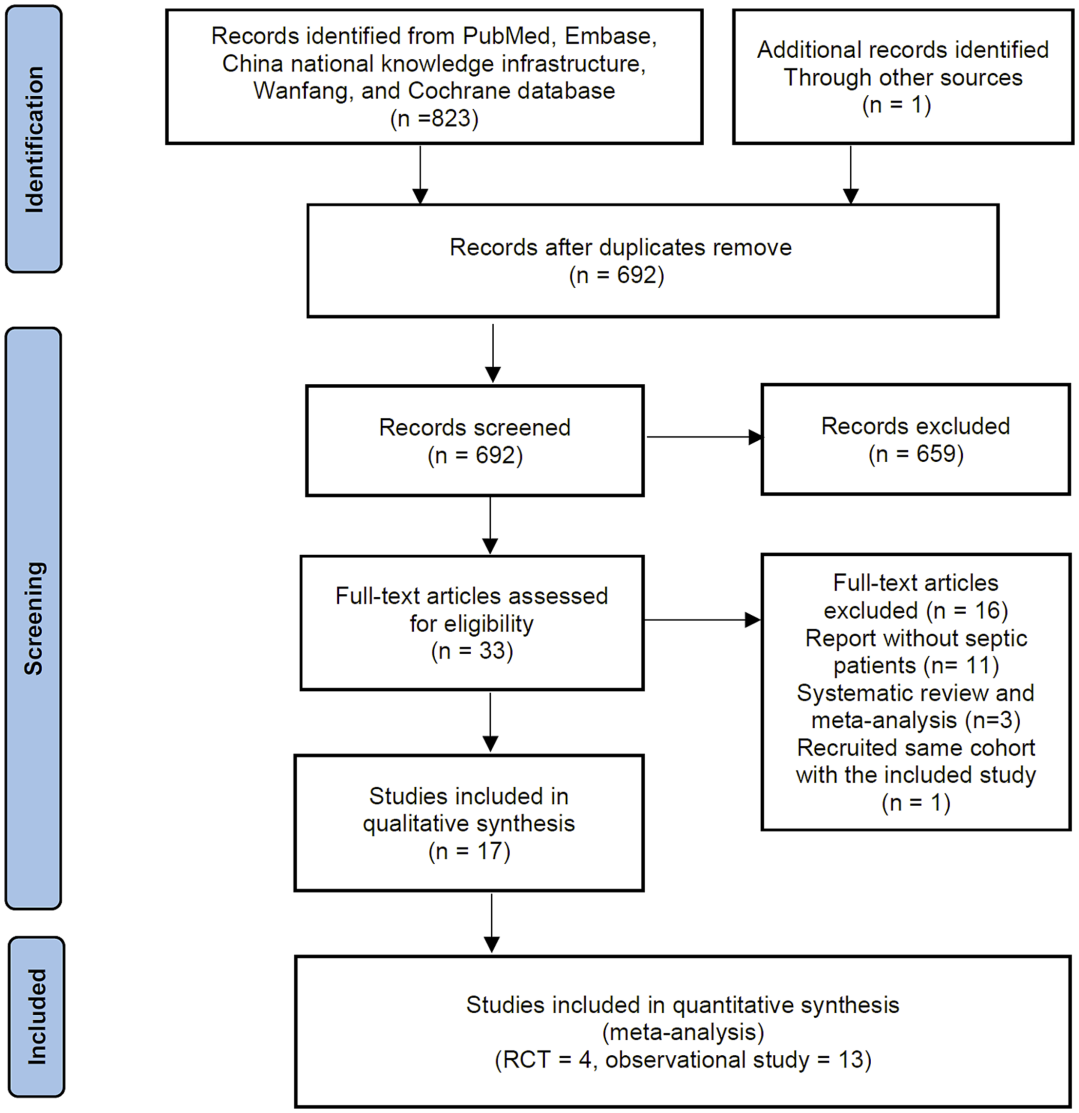
Flowchart of the literature selection process.

Figure 1 presents a flowchart detailing the study selection process. The initial search identified 824 records from online databases and other sources. After removing duplicates and screening the titles and abstracts, 33 references were suitable for full-text review. Ultimately, 17 studies involving a total of 5,062 patients met the eligibility criteria and were included in the final meta-analysis (15–20, 27–37). Among these, four were RCTs, and 13 were retrospective cohort studies.

### Study characteristics and quality assessment

Table 1 summarizes the key information and characteristics of the included studies. All studies were conducted in Japan (*n* = 7) and China (*n* = 10), published between 2004 and 2025. In these studies, 2,391 patients were assigned to the sivelestat group, while 2,671 patients were in the control group. Among the studies, 10 examined unselective septic patients, whereas seven focused on specific septic populations, including pneumonia (*n* = 5) (15, 19, 28, 29, 36), aspiration (*n* = 1) (27), and surgery (*n* = 1) (35). Other characteristics, such as participants’ gender and age, baseline PaO_2_/FiO_2_ ratios, percentage of MV, APACHE II score, and details of the sivelestat protocol used, showed no differences between the sivelestat and control groups.

**Table 1:** Characteristics of the included studies.

Study	Country	Design	Population	Sample		Age, year		Male,%		SIVE regimen, Mg/kg/h	Mean PaO_2_/FiO_2_ at baseline, mmHg		Mortality rate, %
				SIVE	C	SIVE	C	SIVE	C		SIVE	C	
Che 2024	China	R, SC	COVID-19	45	65	77	88	71.7	84.6	NA	157.0	202.0	69.1
Endo 2006	Japan	RCT, SC	Sepsis	13	13	NA	NA	NA	NA	0.2 mg/kg/h, for 14 d	NA	NA	11.5
Gao 2021	China	R, SC	Sepsis	60	80	56.2	57.9	65	72.3	0.2 mg/kg/h, for 14 d	129.25	144.3	5.7
Hayakawa 2010	Japan	P, SC	Sepsis	34	133	59.4	54.1	70.6	56.4	0.2 mg/kg/h, for 14 d	89.8	128.9	22.8
Hayakawa 2011	Japan	RCT, SC	Aspiration	23	21	71.7	63.6	73.9	42.9	0.2 mg/kg/h, for 14 d	118.1	146.2	20.5
Kishimoto 2017	Japan	R, database	Pneumonia	1,516	1,516	72.6	73.3	71.4	72.4	0.2 mg/kg/h, for 14 d	NA	NA	29.0
Li 2025	China	R, MC	COVID-19	79	79	72	72	64.6	65.8	0.2 mg/kg/h, for 14 d	163.4	174.0	22.2
Luo 2023	China	R, SC	COVID-19	35	70	69	71	65.7	65.7	NA	132	136	60.0
Lv 2024	China	RCT, SC	Sepsis	35	35	58.5	56.7	54.3	60.0	0.2 mg/kg/h, for 14 d	NA	NA	NA
Ma 2025	China	R, SC	Sepsis	86	41	72	67	73.3	87.8	0.2 mg/kg/h, for 14 d	111	98	26.0
Miyoshi 2013	Japan	R, MC	Sepsis	70	40	73	71	57.5	68.6	0.2 mg/kg/h, for 14 d	142.9	174.1	40.9
Qi 2023	China	R, SC	Sepsis	70	71	62	62	64.3	70.4	NA	189.0	245.0	15.6
Tamakuma 2004	Japan	RCT, SC	Sepsis	113	108	59.5	56.1	76.1	75.9	0.2 mg/kg/h, for 14 d	NA	NA	24.9
Tsuboko 2012	Japan	R, SC	surgery	34	15	73	69	79.4	66.7	0.2 mg/kg/h, for 14 d	171	182	18.4
Wang 2024	China	R, SC	COVID-19	102	306	73.3	72.9	60.8	53.6	0.2 mg/kg/h, for 14 d	NA	NA	11.8
Wu 2025	China	RCT,	Sepsis	34	36	61.2	56.5	64.7	63.9	0.2 mg/kg/h, for 14 d	136.0	161.0	18.6
Xu 2024	China	R, SC	Sepsis	42	42	58.4	59	61.9	57.1	0.2 mg/kg/h, for 7 d	76.92	78.16	44.0

C, control; MC, multicentre; NA, not available; P, prospective; PaO2/FiO2, partial pressure of oxygen/fraction of inspiration oxygen; RCT, randomized controlled trials; SC, single-center; SIVE, sivelestat.

The quality of RCTs and observational studies were summarized in Supplementary files 2, 3, respectively.

### Mortality

**Figure 2:**
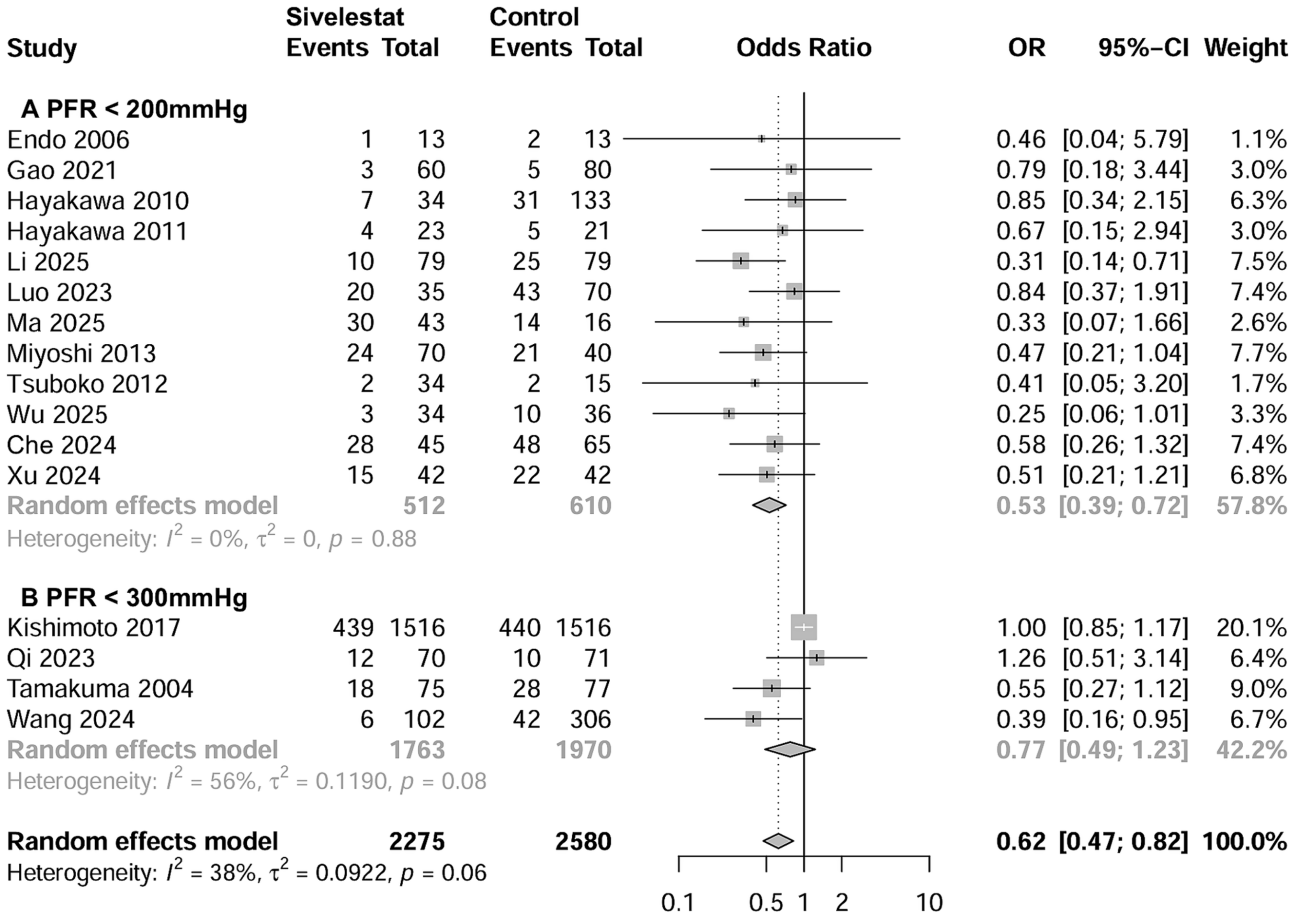
Forest plots of sivelestat on mortality in ARDS patients with PaO_2_/FiO_2_ < 200 mmHg **(A)** and with PaO_2_/FiO_2_ < 300 mmHg **(B)**.

**Figure 3:**
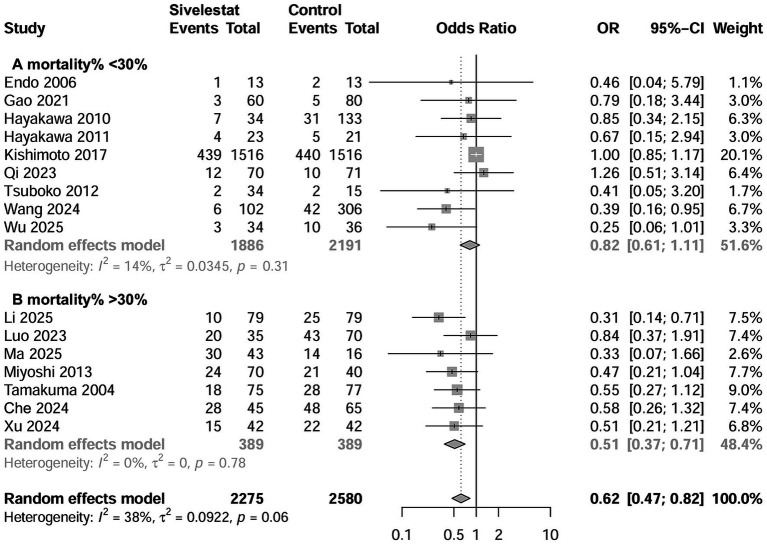
Forest plots of sivelestat on mortality in ARDS patients with mortality rate >30% **(A)** and with mortality rate <30% **(B)**.

A total of 16 studies involving 4,992 patients reported the outcome regarding mortality (15–20, 27–29, 31–37), with a mean mortality rate of 30.1% (1,373 out of 4,992). The pooled results indicated that sivelestat significantly reduced the risk of mortality (OR = 0.62; 95% CI, 0.47–0.82; *I*^2^ = 39%; Figure 2) compared to controls. Sensitivity analyses explored the source of heterogeneity and yielded consistent results with the combined results (Supplementary file 4). Meta-regression showed that differences in initial PaO_2_/FiO_2_ and risk of mortality significantly influence the effectiveness of sivelestat interventions (Supplementary file 5). Specifically, subsequent subgroup analyses revealed a significant reduction in mortality in septic ARDS patients with a PaO_2_/FiO_2_ < 200 mmHg (OR = 0.53, 95% CI 0.39–0.72; *I*^2^ = 0%; Figure 2A; Table 2) and in patients with a mean mortality rate >30% (OR = 0.518, 95% CI 0.37–0.71; *I*^2^ = 0%; Figure 3; Table 2). We also found similar subgroup results and eliminated heterogeneity (*I*^2^ = 0%;) in our *post hoc* analysis by setting the cutoffs for mean mortality rate at 25 and 20% (Supplementary file 6). Funnel plots assessing mortality indicated no publication bias among the included studies (Supplementary file 7).

**Table 2:** Subgroup analyses of the effect of sivelestat on mortality.

Study characteristics	Studies number	Patient number	Event in sivelestat group	Event in control group	Odds ratio (95% CI)	I2
All included studies	16	4,855	157 of 2,275 (15.3%)	228 of 2,580 (30.0%)	0.40 (0.30, 0.54)	38%
Sample size ≥100	10	4,523	567 of 2086 (27.2%)	693 of 2,437 (28.4%)	0.67 (0.49, 0.92)	48%
Sample size <100	6	332	55 of 189 (29.1%)	55 of 143 (38.5%)	0.45 (0.22, 0.91)	0
RCTs	3	248	22 of 122 (18.0%)	40 of 126 (31.7%)	0.47 (0.26, 0.86)	0%
Non-RCTs	13	4,607	600 of 2,153 (27.9%)	708 of 2,454 (28.9%)	0.66 (0.49, 0.88)	38%
Mortality% > 30	7	778	145 of 389 (37.3%)	201 of 389 (51.7%)	0.51 (0.37, 0.71)	0%
Mortality% < 30	9	4,077	477 of 1886 (25.3%)	547 of 2,191 (25.0%)	0.82 (0.61, 1.11)	14%
Initial PaO2/FiO2 < 300 mmHg	4	3,733	475 of 1763 (26.9%)	520 of 1970 (26.4%)	0.77 (0.49, 1.23)	56%
Initial PaO2/FiO2 < 200	12	1,122	157 of 287 (54.7%)	228 of 302 (75.5%)	0.53 (0.39, 0.72)	14%

ICU, intensive care unit; NG, nasogastric tube; NJ, nasojejunal tube; PEG, percutaneous gastrostomy; RCT, randomized controlled trials.

Additionally, six studies used regression analyses to adjust for the confounding factors affecting mortality (15, 20, 28, 29, 32, 36). When pooled, the adjusted hazard risk (HR) from meta-analysis demonstrated that the use of sivelestat was associated with a significantly reduced mortality rate (HR = 0.48; 95% CI 0.28–0.82; Supplementary file 8).

### Secondary outcomes

**Figure 4:**
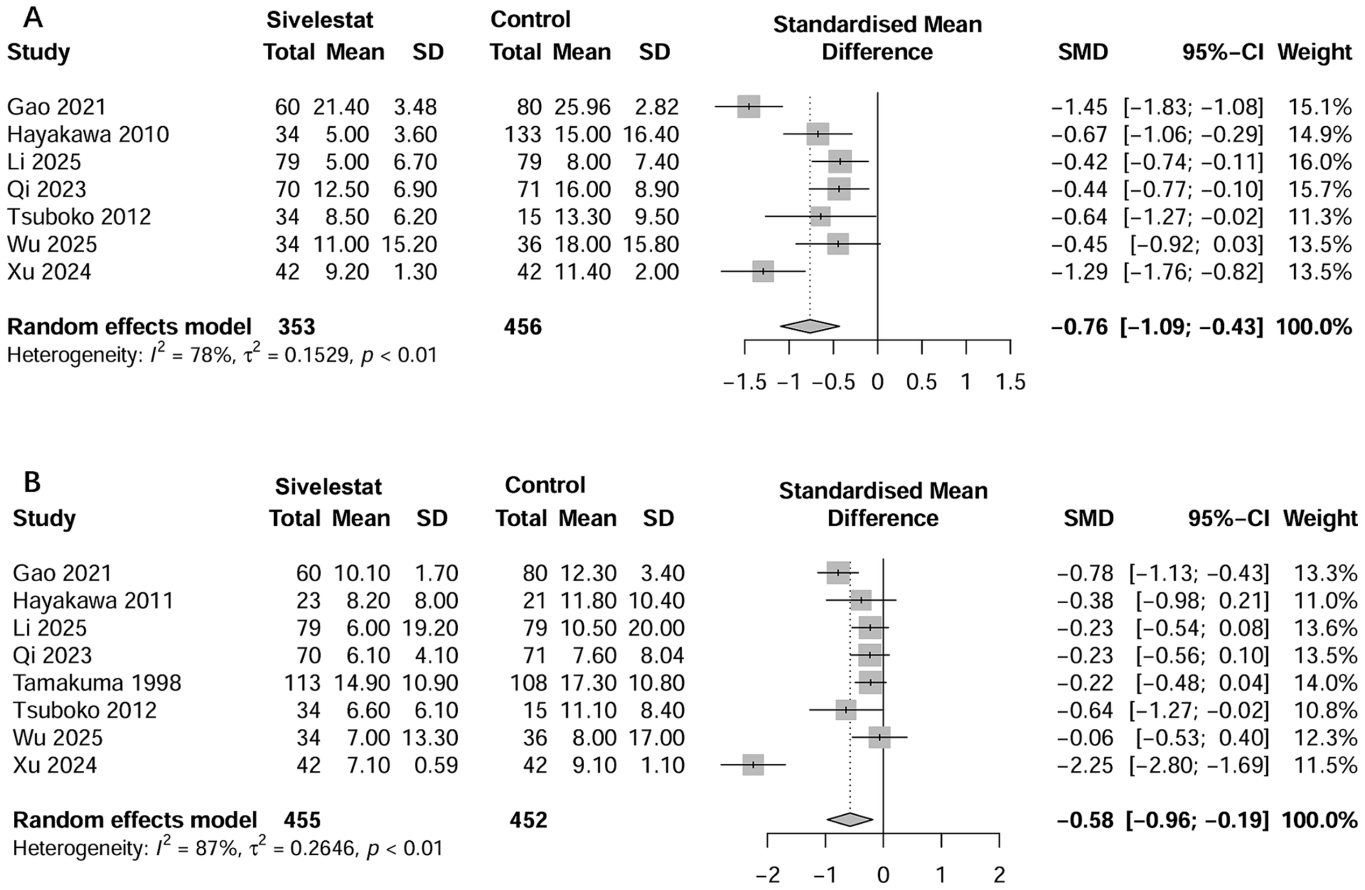
Forest plots of sivelestat on length of stay in ICU **(A)** and duration of mechanical ventilation **(B)** in ARDS patients.

**Figure 5:**
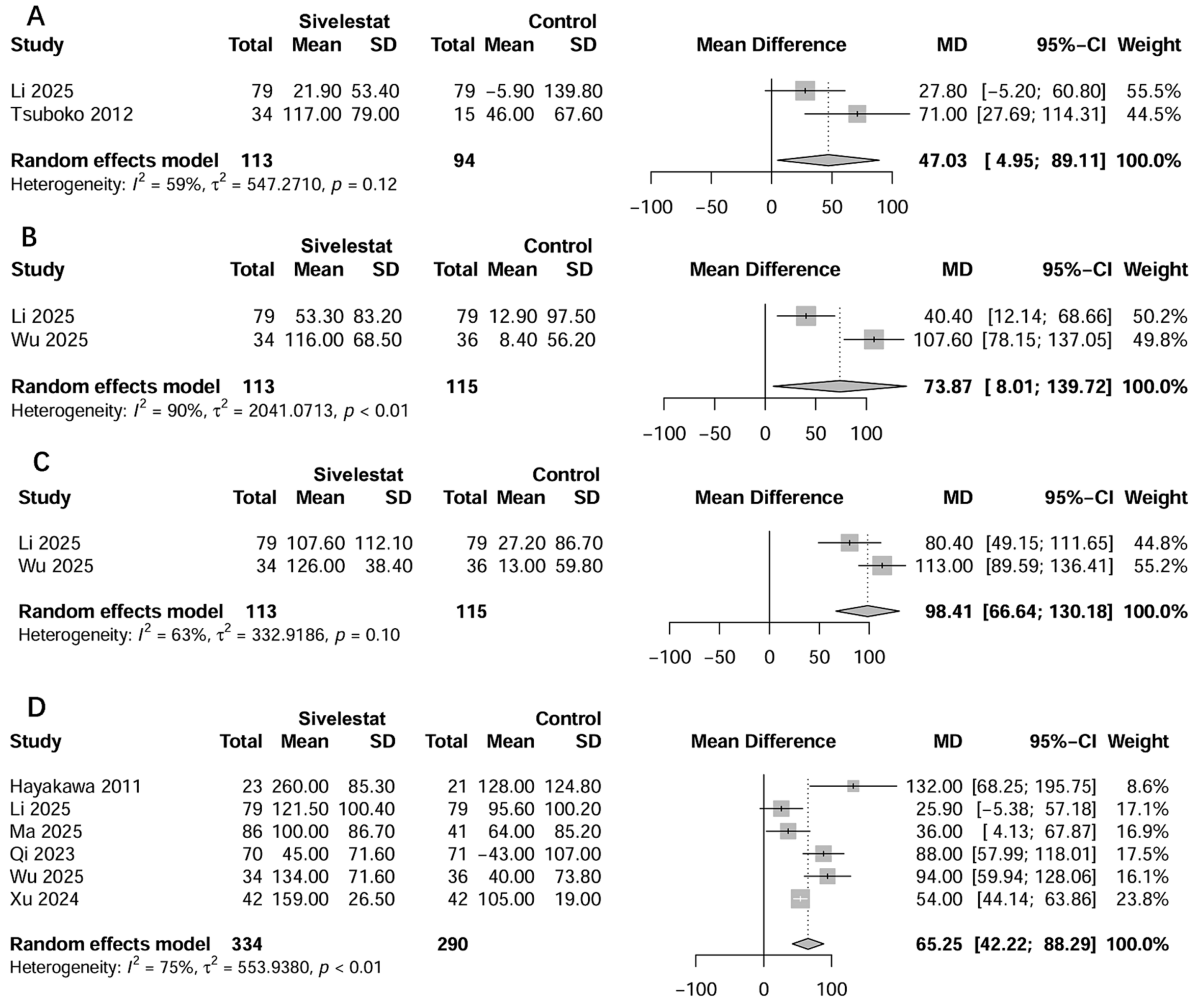
Forest plots of sivelestat on changes in PaO_2_/FiO_2_ at 1 day **(A)**, 3 days **(B)**, 5 days **(C)**, and 7 days **(D)** after treatment.

Seven studies examined the outcome of ICU LOS (16–20, 33, 35). The pooled results showed that use of sivelestat significantly reduced ICU LOS (standard mean difference [SMD] = −0.76 days; 95% CI, −1.09 to −0.43; Figure 4A). Eight studies reported on the outcome of the duration of MV (16–20, 33–35), and the pooled result indicated sivelestat treatment significantly reduced the duration of MV (SMD = -0.58 days; 95% CI, −0.96 to −0.19; Figure 4B). A total of 14 studies described the effect of sivelestat on the ∆PaO_2_/FiO_2_ after treatment; however, only eight studies provided data suitable for combination (16, 18–20, 27, 31, 33, 35). When these studies were pooled, sivelestat was associated with a significantly increase in ∆PaO_2_/FiO_2_ at different time points: 1 day (MD = 47.0 mmHg; 95% CI, 4.95–8.91) (19, 35), 3 days (MD = 73.8 mmHg; 95% CI, 8.01–139.7) (19, 20), 5 days (MD = 98.4 mmHg; 95% CI, 66.6–130.2) (19, 20), and 7 days (MD = 65.3 mmHg; 95% CI, 42.2–88.3) (18–20, 27, 31, 33) after treatment (Figure 5). In addition, AEs varied among the included studies (Supplementary file 9). Hematological abnormalities, live and kidney injury, and total AEs were reported by at least two studies. When pooled, no significant differences were found between the sivelestat and control groups (*p* values ranged from 0.14 to 0.40; Supplementary file 9).

## Discussion

In the current meta-analysis, we included 17 studies to thoroughly evaluate the impact of sivelestat on patients with septic ARDS. Our results indicated that sivelestat therapy significantly reduced all-cause mortality (OR = 0.61; 95% CI 0.51–0.73). These findings were further supported by sensitivity analyses and subgroup analyses of patients with a PaO_2_/FiO_2_ < 200 mmHg and those with a mortality rate >30%. A similar outcome was observed when pooling results from adjusted regression analyses. Additionally, sivelestat also improved ∆PaO_2_/FiO_2_ after treatment, serum inflammatory factors, ICU LOS, and duration of MV.

### Sivelestat technology research

NE is the primary enzyme found in nitrogenous granules within the cytoplasm of neutrophils. Excessive activity of NE can cause tissue damage and re-modeling in ARDS, making it a potential target for treatment (9). NE inhibitors, particularly sivelestat, have shown protective effects against ARDS in various lung injury models (38, 39), prompting researchers and clinicians to explore their use in treating this condition.

Early observational studies suggested that sivelestat significantly reduced the duration of MV and ICU LOS, improved lung function, facilitated weaning from MV, and even decreased mortality in ARDS patients (34). However, these benefits were not confirmed by the international multicenter RCT published in 2004, also known as STRIVE trial (12), which found that sivelestat did not improve 28-day mortality rate in ARDS patients. As a result, these negative results diminished clinical enthusiasm for using sivelestat in this patient population. The failure of the STRIVE trial (12) may be due to the inclusion of patients who met the diagnostic criteria established by the 1994 American-European Consensus Conference on ARDS (23), without adequately excluding those with other underlying conditions. Additionally, early termination of the STRIVE trial may have influenced the negative outcomes (23). Two subsequent meta-analyses based on RCTs also failed to confirm the clinical benefits of sivelestat for ARDS (13, 14). However, both meta-analyses included only a limited number of RCTs, and aside from the STRIVE trial, most were small-sample studies. Notably, the negative results of the meta-analyses were mainly driven by the STRIVE trial, which contributed 85% (13) and 98.8% (14) of the weight, respectively. Furthermore, none of these studies clarified why the significant improvement in patient oxygenation with sivelestat did not translate into better clinical outcomes.

Conversely, the latest ARDS guidelines have proposed efficacy benefits based on ARDS phenotypes (7). For example, patients with high-inflammatory phenotypes have higher 90-day mortality compared to those with low-inflammatory phenotypes (40). Therefore, identifying subgroups of ARDS patients most likely to benefit from specific treatments could facilitate more targeted therapeutic approaches and address these issues.

Given the intense inflammatory response involved in the pathogenesis of sepsis, it is worth exploring whether ARDS occurring alongside sepsis responds better to sivelestat treatment. Therefore, the current meta-analysis included ARDS patients with sepsis and comprehensively assessed the impact of sivelestat on mortality in this population. This analysis included comparisons between sivelestat and control groups, as well as exploring the linear relationship between sivelestat administration and risk of mortality. Additionally, our study featured a large sample size of over 5,000 cases, providing sufficient statistical power for subgroup and sensitivity analyses based on various potential impact factors. Finally, the secondary outcomes showed encouraging information that further supports the robustness of our primary findings.

### Explanation of our research results

This study found that patients with moderate to severe septic ARDS appear to benefit more from sivelestat therapy. This finding contradicts previous beliefs that sivelestat may be more appropriate for patients with mild ARDS. For example, clinical studies reporting excellent results with sivelestat have selected ARDS patients with a lung injury score (LIS) of less than 2.5 (12, 41). Specifically, sivelestat improved mortality rate and duration of MV in a subgroup of patients from the STRIVE trial who had a mean LIS of less than 2.5 and exhibited a systemic inflammatory response syndrome (12). However, these earlier findings were based on the results of various heterogeneous populations and subgroup analyses. Interestingly, several previous large RCTs of ARDS have confirmed that patients with moderate to severe ARDS, rather than mild ARDS, can benefit from interventions such as prone position ventilation (42) and neuromuscular blocking agents use (43). Our findings regarding septic ARDS support this perspective. We hypothesize that in patients with sepsis, excessive NE activation is more likely to occur in moderate to severe ARDS, and that the heightened inflammatory state in these cases might respond better to NE inhibition. In contrast, NE was not significantly activated in mild ARDS, suggesting that NE inhibition could potentially suppress normal NE function in these cases. Our current analyses supported this hypothesis.

On the other hand, variations in defining ARDS severity can lead to different results. In our study, only five of the included studies reported the LIS (17–19, 27, 37). We found that sivelestat significantly improved oxygenation, LIS, duration of MV, length of stay in ICU, and even mortality, regardless of whether the mean LIS was less than 2.5 (17, 19, 27) or greater than 2.5 (18). Another study noted no significant correlation between LIS and PMN-E levels or PaO_2_/FiO_2_ at the time of ALI diagnosis (37). Conversely, PaO_2_/FiO_2_ was used in almost all of the included studies. Therefore, we adopted this guideline-defined indicator of ARDS severity, which has higher statistical efficacy, to reveal the effect of sivelestat on ARDS of different severity.

Meanwhile, we classified ARDS severity based on mortality risk. We observed that ARDS populations with higher mortality appear to benefit more from sivelestat therapy. This may be because it is difficulty to identify positive results in populations with a lower mortality risk. Nevertheless, mortality rates based on various thresholds (20, 25, and 30%) highlight the source of heterogeneity among the included studies, supporting the notion that the efficacy of sivelestat varies with the severity of ARDS.

Additionally, most included studies have demonstrated that sivelestat improved the oxygenation index. In this study, we focused on specific studies where detailed data were available and observed significant improvements in the oxygenation index on days 1, 3, 5, and 7 after treatment. Previous meta-analyses (13, 14), which incorporated only two (14) or four studies (13), yielded different results, likely due to their limited studies. Unlike those earlier analyses that relied on post-treatment values, we investigated the changes in oxygenation index before and after treatment, providing a more accurate representation of actual changes in oxygenation. Our findings also revealed that sivelestat significantly reduced the duration of MV by approximately 2.5 days and shortened ICU stay by about 5 days for septic ARDS patients. These notable results were accompanied by improved oxygenation, which is critical, as prolonged MV and extended hospital stays are associated with poor prognosis for ICU patients.

### Limitations

Our meta-analysis has several limitations. First, the time span of the included literature was too broad, with two guideline updates during the period and ongoing development in ARDS treatment strategies. Second, some of the studies were retrospective cohort studies, limiting our ability to establish causality. Additionally, our analyses included only four RCTs (27, 34, 36, 37), three of which had sample sizes of fewer than 100 participants. Third, including studies focused on different sites of infection, such as COVID-19, aspiration, and abdominal sepsis, may have impacted the accuracy of the findings. Fourth, caution is necessary when interpreting outcomes for individual subgroups of patients, given the insufficient population sizes and sample numbers within these subgroups. Fifth, usual treatments for ARDS patients, such as anti-inflammatory and anti-infection drugs, were implemented without clear specifications and documentation for ARDS cluster therapy. Even with the inclusion of propensity score-matched data, residual confounding factors could not be entirely ruled out. There was also a lack of long-term outcomes such as lung function and quality of life in the included studies. Sixth, we based the oxygenation index threshold on 200 mmHg, primarily considering the definitions of the subgroups and guidelines from the included studies. However, some studies suggest that a threshold of 150 mmHg may better reflect disease severity (42, 43). Seventh, the included studies lacked a uniform dosing regimen and timing of initiation of sivelestat, which limited the direct clinical translation of our conclusions. Finally, the included studies were predominantly from Asian populations, specifically Japan and China. Unlike in Asian countries, there appears to be a lack of interest in developing NE inhibitors for ARDS in non-Asian countries. Potential publication bias may overestimate treatment effects. Therefore, future well-powered, multicenter RCTs should consider applications in different population groups.

## Conclusion

Our analysis indicates that sivelestat reduced the risk of mortality in patients with septic ARDS. Additionally, sivelestat significantly improved the ∆PaO_2_/FiO_2_ ratio after treatment and was associated with a significantly reduction in ICU LOS, duration of MV. Furthermore, there were no differences in adverse events between the sivelestat and control groups. Large, well-designed, multicentre trials are necessary to further confirm the safety and efficacy of sivelestat in this patient population.

## Data Availability

The original contributions presented in the study are included in the article/Supplementary material, further inquiries can be directed to the corresponding author.
