# Supplementary material for: Sivelestat for septic patients with acute respiratory distress syndrome: a systematic review and meta-analysis of a deadly duo
Source: Front Med (Lausanne). 2025 Oct 20;12:1679717. doi: 10.3389/fmed.2025.1679717 (PMC12580385; doi:10.3389/fmed.2025.1679717)
Supplement: Supplementary file 1 [file Data_Sheet_1.docx]

**Sivelestat for septic patients with acute respiratory distress syndrome: a systematic review and meta-analysis of a deadly duo**

**Additional Files**

Additional File 1 PRISMA checklist…………………………………………………………………………………………………………………………………………………………………………………………….…………………………………..…..2

Additional File 2 Assessment of RCTs quality…………………………………………………………………………………………………………………………………………………………………………………….…………….………………....5

Additional File 3 Assessment of observational studies quality………………………………………………………………………………………………………………………………………………………………………………..…………..6

Additional File 4 Sensitivity analysis.………………….…………………………………………………………………………………………………………………………………………….........................................................................….…….7

Additional File 5 Meta regression analyses…………………………………………………………………………………………………………………………………………………………………………………………………………………..…….8

Additional File 6 Subgroup analyses based on mean mortality rate (>25% or <25%) and (>20% or <20%)……………………………….………………………………………………………..………………….…………9

Additional File 7 Funnel plot of comparison: ……………………………….…..………………………………………………………………….…………………………………………………………….…………………………………....………10

Additional File 8 Forest plots of sivelestat on mortality in ARDS patients when pooling adjusted hazard risk (HR) from meta-analysis………………………….………………………………………....…….11

Additional File 9 Adverse events…………………………. …………….…………………………….…………………………………………………………………………………………………………………………….………………………....…….12

**Additional File 1**

**PRISMA 2009 checklist**

| **Section/topic** | **#** | **Checklist item** | **Reported on page #** |
| --- | --- | --- | --- |
| **TITLE** | | |  |
| Title | 1 | Identify the report as a systematic review, meta-analysis, or both. | 1 |
| **ABSTRACT** | | |  |
| Structured summary | 2 | Provide a structured summary including, as applicable: background; objectives; data sources; study eligibility criteria, participants, and interventions; study appraisal and synthesis methods; results; limitations; conclusions and implications of key findings; systematic review registration number. | 3 |
| **INTRODUCTION** | | |  |
| Rationale | 3 | Describe the rationale for the review in the context of what is already known. | 5 |
| Objectives | 4 | Provide an explicit statement of questions being addressed with reference to participants, interventions, comparisons, outcomes, and study design (PICOS). | 5-6 |
| **METHODS** | | |  |
| Protocol and registration | 5 | Indicate if a review protocol exists, if and where it can be accessed (e.g., Web address), and, if available, provide registration information including registration number. | 7 |
| Eligibility criteria | 6 | Specify study characteristics (e.g., PICOS, length of follow-up) and report characteristics (e.g., years considered, language, publication status) used as criteria for eligibility, giving rationale. | 7 |
| Information sources | 7 | Describe all information sources (e.g., databases with dates of coverage, contact with study authors to identify additional studies) in the search and date last searched. | 7 |
| Search | 8 | Present full electronic search strategy for at least one database, including any limits used, such that it could be repeated. | 7-8 and Additional File 2 |
| Study selection | 9 | State the process for selecting studies (i.e., screening, eligibility, included in systematic review, and, if applicable, included in the meta-analysis). | 7 |
| Data collection process | 10 | Describe method of data extraction from reports (e.g., piloted forms, independently, in duplicate) and any processes for obtaining and confirming data from investigators. | 7-8 |
| Data items | 11 | List and define all variables for which data were sought (e.g., PICOS, funding sources) and any assumptions and simplifications made. | 8 |
| Risk of bias in individual studies | 12 | Describe methods used for assessing risk of bias of individual studies (including specification of whether this was done at the study or outcome level), and how this information is to be used in any data synthesis. | 8 |
| Summary measures | 13 | State the principal summary measures (e.g., risk ratio, difference in means). | 8 |
| Synthesis of results | 14 | Describe the methods of handling data and combining results of studies, if done, including measures of consistency (e.g., I^2^) for each meta-analysis. | 9 |

| Risk of bias across studies | 15 | Specify any assessment of risk of bias that may affect the cumulative evidence (e.g., publication bias, selective reporting within studies). | 8 |
| --- | --- | --- | --- |
| Additional analyses | 16 | Describe methods of additional analyses (e.g., sensitivity or subgroup analyses, meta-regression), if done, indicating which were pre-specified. | 9 |
| **RESULTS** | | |  |
| Study selection | 17 | Give numbers of studies screened, assessed for eligibility, and included in the review, with reasons for exclusions at each stage, ideally with a flow diagram. | 10and Figure 1, |
| Study characteristics | 18 | For each study, present characteristics for which data were extracted (e.g., study size, PICOS, follow-up period) and provide the citations. | 10  Table 1, |
| Risk of bias within studies | 19 | Present data on risk of bias of each study and, if available, any outcome level assessment (see item 12). | 10-11 |
| Results of individual studies | 20 | For all outcomes considered (benefits or harms), present, for each study: (a) simple summary data for each intervention group (b) effect estimates and confidence intervals, ideally with a forest plot. | 11 |
| Synthesis of results | 21 | Present results of each meta-analysis done, including confidence intervals and measures of consistency. | 11-12 |
| Risk of bias across studies | 22 | Present results of any assessment of risk of bias across studies (see Item 15). | Additional File 3 |
| Additional analysis | 23 | Give results of additional analyses, if done (e.g., sensitivity or subgroup analyses, meta-regression [see Item 16]). | 11-12  Additional File 4-5 |
| **DISCUSSION** | | |  |
| Summary of evidence | 24 | Summarize the main findings including the strength of evidence for each main outcome; consider their relevance to key groups (e.g., healthcare providers, users, and policy makers). | 13-18 |
| Limitations | 25 | Discuss limitations at study and outcome level (e.g., risk of bias), and at review-level (e.g., incomplete retrieval of identified research, reporting bias). | 17-18 |
| Conclusions | 26 | Provide a general interpretation of the results in the context of other evidence, and implications for future research. | 19 |
| **FUNDING** | | |  |
| Funding | 27 | Describe sources of funding for the systematic review and other support (e.g., supply of data); role of funders for the systematic review. | 20 |

**Additional File 2**

**Assessment of RCTs quality**

| **Study** | **Random sequence generation (selection bias)** | **Allocation concealment (selection bias)** | **Blinding of participants and personnel (performance bias)** | **Blinding of outcome assessment (detection bias)** | **Incomplete outcome data (attrition bias)** | **Selective reporting (reporting bias)** | **Other bias** |
| --- | --- | --- | --- | --- | --- | --- | --- |
| Endo 2006 | low | Unclear | Ucleaar | Ucleaar | low | low | low |
| Lv 2024 | low | low | low | low | low | low | low |
| Tamakuma 2004 | low | low | low | low | low | low | low |
| Wu 2025 | low | low | low | low | low | low | low |

**Additional File 3 Assessment of observational studies quality**

**Quality assessment and overall risk of bias of included studies**

| First author / year | Patient selection | | | | Comparability | Outcome | | | Risk of bias |
| --- | --- | --- | --- | --- | --- | --- | --- | --- | --- |
|  | Representation of the exposed cohort | Selection of the non-exposed cohort | Ascertainment of exposure | Outcome of  interest not  present at start | Comparability of cohorts on the basis of the design or analysis | Assessment  of outcome | Was follow-up long enough for outcomes to occur | Adequacy of follow up of cohorts |  |
| Che 2024 | ★ | ★ | ★ | ☆ | ★★ | ★ | ★ | ★ | 8 |
| Gao 2021 | ★ | ★ | ★ | ☆ | ★ | ★ | ★ | ★ | 7 |
| Hayakawa 2010 | ★ | ★ | ★ | ☆ | ★ | ★ | ★ | ★ | 7 |
| Hayakawa 2011 | ★ | ★ | ★ | ☆ | ★★ | ★ | ★ | ★ | 8 |
| Kishimoto 2017 | ★ | ★ | ★ | ☆ | ★★ | ★ | ★ | ★ | 8 |
| Li 2025 | ★ | ★ | ★ | ☆ | ★ | ★ | ★ | ★ | 7 |
| Luo 2023 | ★ | ★ | ★ | ☆ | ★ | ★ | ★ | ★ | 7 |
| Ma 2025 | ★ | ★ | ★ | ☆ | ★★ | ★ | ★ | ★ | 8 |
| Miyoshi 2013 | ★ | ★ | ★ | ☆ | ★★ | ★ | ★ | ★ | 8 |
| Qi 2023 | ★ | ★ | ★ | ☆ | ★ | ★ | ★ | ★ | 7 |
| Tsuboko 2012 | ★ | ★ | ★ | ☆ | ★★ | ★ | ★ | ★ | 8 |
| Wang 2024 | ★ | ★ | ★ | ☆ | ★★ | ★ | ★ | ★ | 8 |
| Xu 2024 | ★ | ★ | ★ | ☆ | ★★ | ★ | ★ | ★ | 8 |

**Additional File 4 Sensitivity analysis**

> metainf (metaM)

Influential analysis (Random effects model)

OR 95%-CI p-value tau^2 tau I^2

Omitting Endo 2006 0.6219 [0.4686; 0.8254] 0.0010 0.1009 0.3176 41.6%

Omitting Gao 2021 0.6134 [0.4597; 0.8185] 0.0009 0.1050 0.3240 42.1%

Omitting Hayakawa 2010 0.6030 [0.4483; 0.8111] 0.0008 0.1098 0.3314 42.1%

Omitting Hayakawa 2011 0.6169 [0.4625; 0.8226] 0.0010 0.1041 0.3226 41.9%

Omitting Kishimoto 2017 0.5557 [0.4301; 0.7180] < 0.0001 0.0000 0.0000 0.0%

Omitting Li 2025 0.6918 [0.5390; 0.8879] 0.0038 0.0468 0.2164 23.3%

Omitting Luo 2023 0.6009 [0.4454; 0.8106] 0.0009 0.1119 0.3345 42.1%

Omitting Ma 2025 0.6342 [0.4799; 0.8381] 0.0014 0.0909 0.3015 38.8%

Omitting Miyoshi 2013 0.6395 [0.4810; 0.8504] 0.0021 0.0887 0.2978 36.4%

Omitting Qi 2023 0.5894 [0.4403; 0.7891] 0.0004 0.1020 0.3194 40.3%

Omitting Tamakuma 2004 0.6275 [0.4678; 0.8418] 0.0019 0.0990 0.3146 38.3%

Omitting Tsuboko 2012 0.6251 [0.4713; 0.8291] 0.0011 0.0986 0.3141 40.9%

Omitting Wang 2024 0.6512 [0.4944; 0.8579] 0.0023 0.0780 0.2793 33.9%

Omitting Wu 2025 0.6511 [0.4979; 0.8515] 0.0017 0.0747 0.2732 34.0%

Omitting Xiao 2024 0.6220 [0.4636; 0.8344] 0.0015 0.1029 0.3208 40.1%

Omitting Xu 2024 0.6313 [0.4733; 0.8421] 0.0018 0.0956 0.3092 38.6%

Pooled estimate 0.6232 [0.4729; 0.8212] 0.0008 0.0922 0.3036 38.0%

Details on meta-analytical method:

- Mantel-Haenszel method

- DerSimonian-Laird estimator for tau^2

**Additional File 5:**

**Meta regression**

> metareg(metaM, ~severity+ran+year+sample)

Mixed-Effects Model (k = 15; tau^2 estimator: DL)

tau^2 (estimated amount of residual heterogeneity): 0 (SE = 0.0945)

tau (square root of estimated tau^2 value): 0

I^2 (residual heterogeneity / unaccounted variability): 0.00%

H^2 (unaccounted variability / sampling variability): 1.00

R^2 (amount of heterogeneity accounted for): 100.00%

Test for Residual Heterogeneity:

QE(df = 10) = 8.6904, p-val = 0.5617

Test of Moderators (coefficients 2:5):

QM(df = 4) = 14.1478, p-val = 0.0068

Model Results:

estimate se zval pval ci.lb ci.ub ​

intrcpt -0.6811 1.0378 -0.6562 0.5117 -2.7152 1.3530

severity -0.5727 0.2257 -2.5370 0.0112 -1.0152 -0.1303 *

ran 0.6572 0.4017 1.6360 0.1018 -0.1301 1.4445

year -0.1068 0.3027 -0.3527 0.7243 -0.7000 0.4865

sample 0.0640 0.3781 0.1693 0.8656 -0.6770 0.8050

**-----------------------------------------------------------------------------------------------------------------------**

**> metareg(metaM, ~dead%+ran+year+sample)**

Mixed-Effects Model (k = 16; tau^2 estimator: DL)

tau^2 (estimated amount of residual heterogeneity): 0 (SE = 0.0889)

tau (square root of estimated tau^2 value): 0

I^2 (residual heterogeneity / unaccounted variability): 0.00%

H^2 (unaccounted variability / sampling variability): 1.00

R^2 (amount of heterogeneity accounted for): 100.00%

Test for Residual Heterogeneity:

QE(df = 11) = 9.5681, p-val = 0.5696

Test of Moderators (coefficients 2:5):

QM(df = 4) = 14.5949, p-val = 0.0056

Model Results:

estimate se zval pval ci.lb ci.ub ​

intrcpt -2.4415 0.7908 -3.0875 0.0020 -3.9914 -0.8916 **

dead 0.4912 0.2031 2.4188 0.0156 0.0932 0.8892 *

ran 0.1897 0.3830 0.4953 0.6204 -0.5609 0.9403

year 0.0540 0.2789 0.1935 0.8466 -0.4927 0.6006

sample 0.4676 0.3115 1.5013 0.1333 -0.1429 1.0782

**Additional File 6**


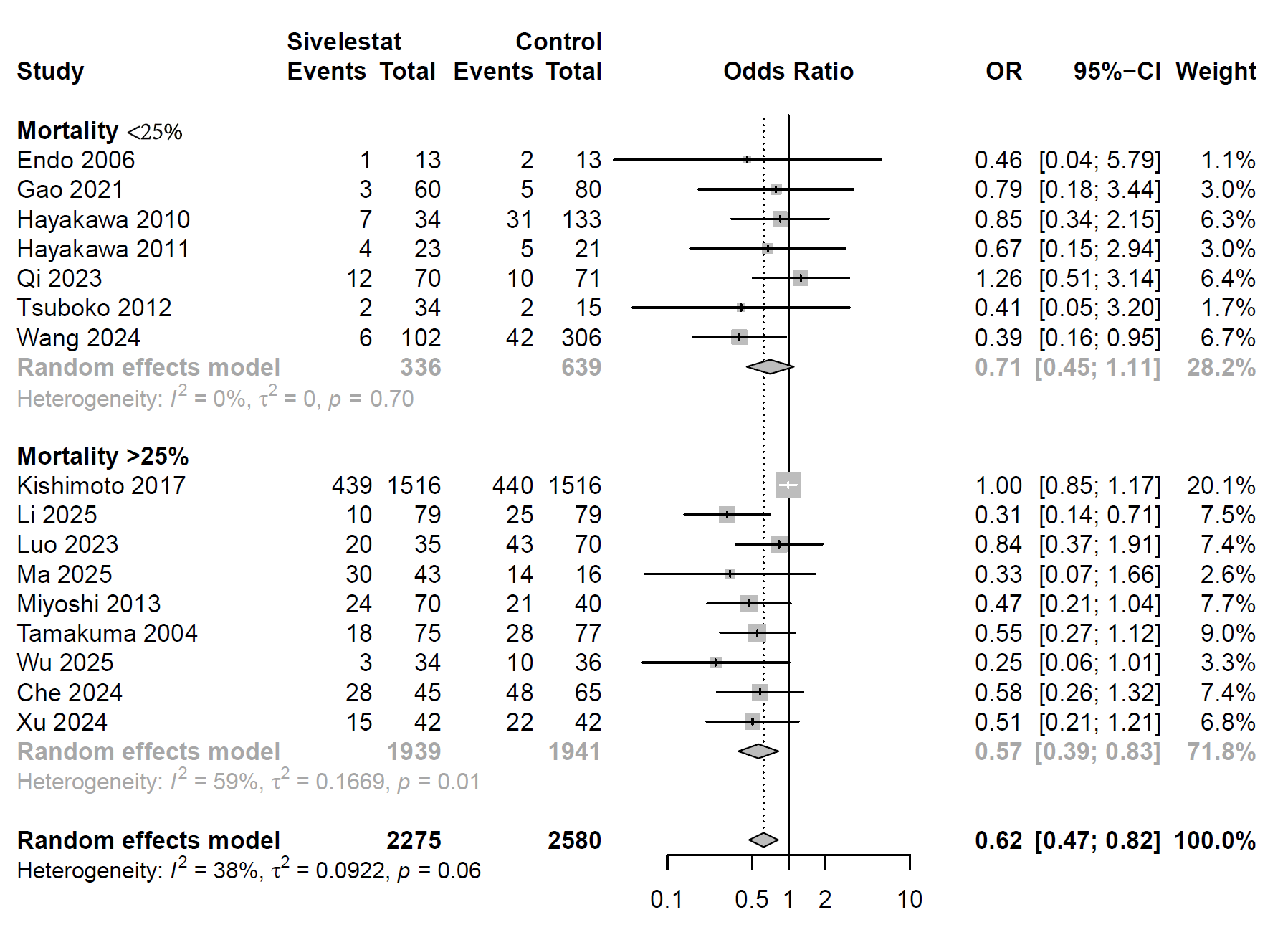


**Forest plots of sivelestat on mortality in ARDS patients with mortality rate >25% and with mortality rate <25%.**


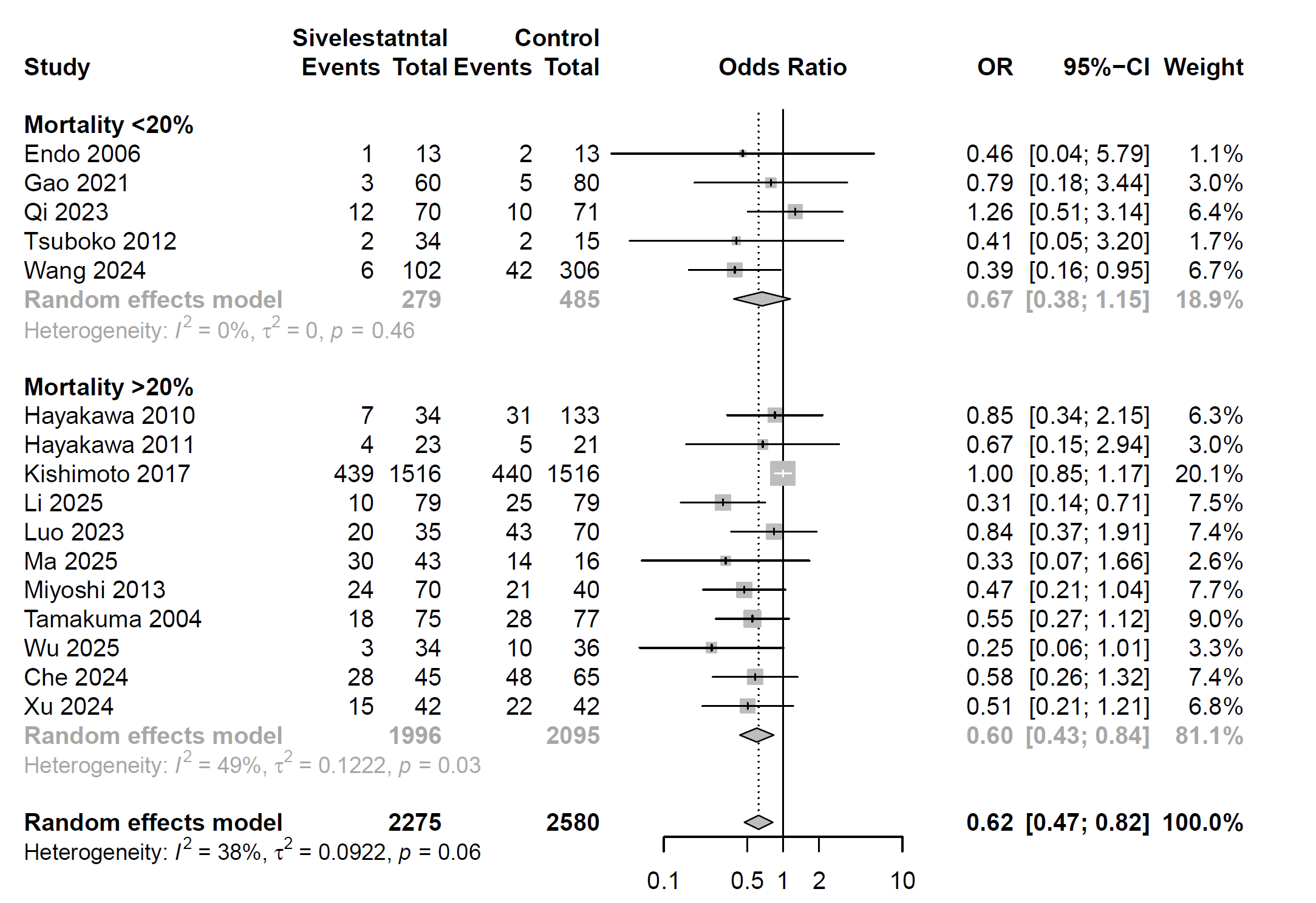


**Forest plots of sivelestat on mortality in ARDS patients with mortality rate >20% and with mortality rate <20%.**

**Additional File 7**

**Funnel plot of comparison: Mortality**


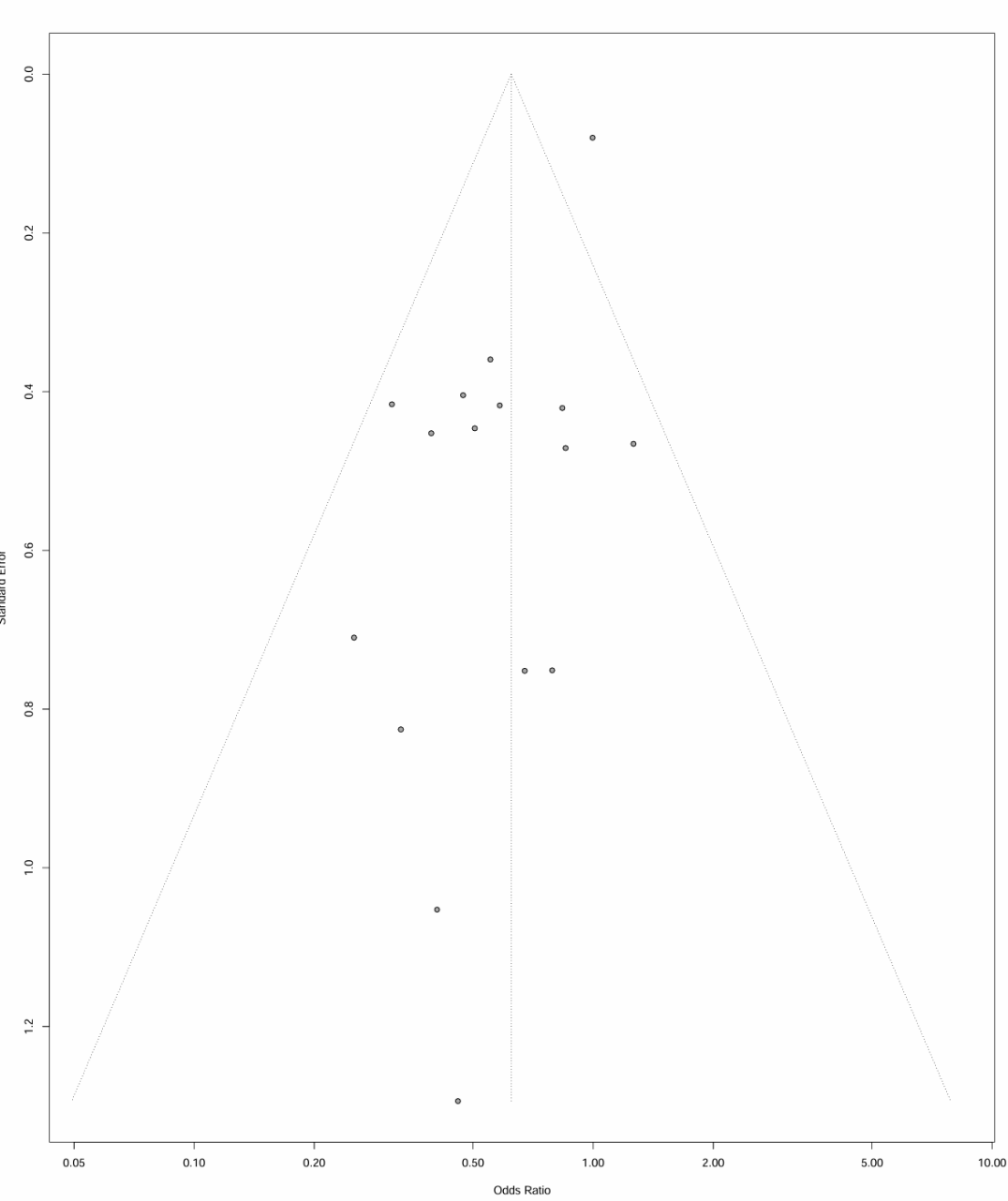


**Additional File 8**

**Forest plots of sivelestat on mortality in ARDS patients when pooling adjusted hazard risk (HR) from meta-analysis**


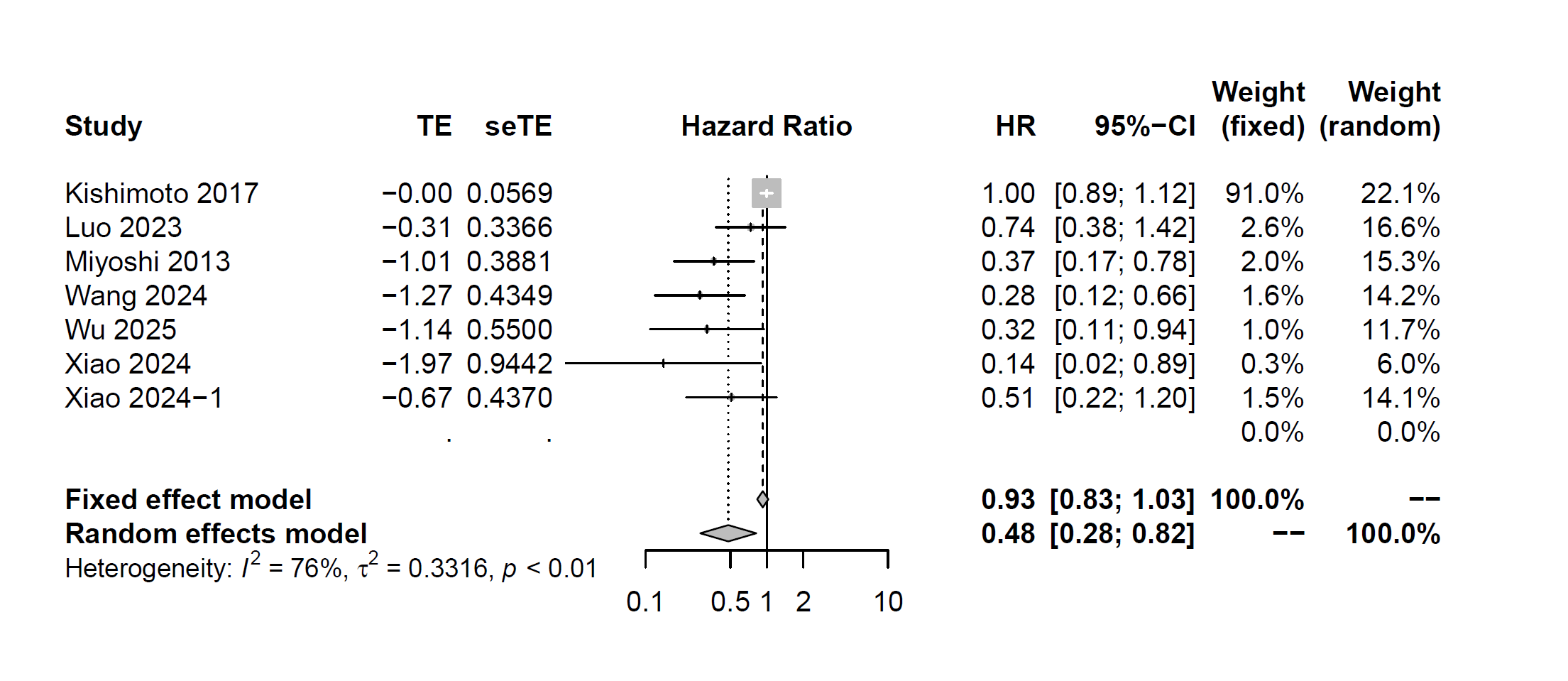


**Additional File 9**

**Summary definitions of adverse events in each included study.**

| **Study** | **Adverse events** | | **Pooled Data available** |
| --- | --- | --- | --- |
| Che 2024 | There were no significant differences between the two groups in terms of liver insufficiency (increased ALT, AST, TBIL, and DBIL) or renal insufficiency (increased SU and sCr, and a decreased GFR) 1-7 days after surgery (all P>0.05). | | No |
| Li 2025 | **Total adverse events** | 3/79; 1/79; |  |
| Wu 2025 | Type of adverse event | Sivelestat vs. Control (n,) | Yes |
|  | Hematological Abnormalities | 2/34; 2/36; |  |
|  | Abnormal liver function | 1/34; 2/36; |  |
|  | Hyperuricemia | 0/34; 1/36; |  |
|  | Hyperlactacidemia | 0/34; 1/36; |  |
| Xu 2024 | Type of adverse event | Sivelestat vs. Control (n) | Yes |
|  | Nausea and vomiting | 4/42 9.52%; 2/42 4.76% |  |
|  | Rash and itching | 1/42 2.38%; 2/42 4.76% |  |
|  | Liver and kidney dysfunction | 1/42 2.38%; 0/42 0.00% |  |
|  | Blood platelet disorder | 1/42 2.38%; 0/42 0.00% |  |
|  | Hemocytopenia | 1/42 2.38%; 1/42 2.38% |  |
|  | **Total adverse events** | 8/42 19.05%; 5/42 11.90% |  |
| Wang 2024 | Type of adverse event | Sivelestat vs. Control (n, %) | Yes |
|  | Liver-injury | 34/102 33.3%; 727/2352 30.9% |  |
|  | Kidney-injury | 28/102 27.5%; 603/2352 25.6% |  |
|  | Urine protein | 6/102 5.90%; 143/2352 6.1% |  |
|  | Leukocytopenia | 11/102 10.8%; 244/2352 10.4% |  |
|  | Thrombopenia | 21/102 20.6%; 349/2352 14.8% |  |

1. Hematological Abnormalities: (3 studies, OR=1.36, 95CI% 0.90-2.05; *I*^2^=0%; P=0.14).

2. Total adverse events (2 studies, OR=2.00, 95CI% 0.29-5.78; *I*^2^=0%; P=0.20).

3. Liver and kidney injury (3 studies, OR=1.18, 95CI% 0.80-1.76; *I*^2^=0%; P=0.40).
